# Casein Kinase-2 Interacting Protein-1 Regulates Physiological Cardiac Hypertrophy *via* Inhibition of Histone Deacetylase 4 Phosphorylation

**DOI:** 10.3389/fphys.2021.678863

**Published:** 2021-06-15

**Authors:** Yinlong Zhao, Shukuan Ling, Guohui Zhong, Yuheng Li, Jianwei Li, Ruikai Du, Xiaoyan Jin, Dingsheng Zhao, Zizhong Liu, Guanghan Kan, Yan-Zhong Chang, Yingxian Li

**Affiliations:** ^1^Key Laboratory of Molecular and Cellular Biology of Ministry of Education, College of Life Science, Hebei Normal University, Shijiazhuang, China; ^2^State Key Laboratory of Space Medicine Fundamentals and Application, China Astronaut Research and Training Center, Beijing, China; ^3^School of Aerospace Medicine, Fourth Military Medical University, Xi’an, China

**Keywords:** CKIP-1, swimming, physiological cardiac hypertrophy, pathological cardiac remodeling, HDAC4

## Abstract

Different kinds of mechanical stimuli acting on the heart lead to different myocardial phenotypes. Physiological stress, such as exercise, leads to adaptive cardiac hypertrophy, which is characterized by a normal cardiac structure and improved cardiac function. Pathological stress, such as sustained cardiac pressure overload, causes maladaptive cardiac remodeling and, eventually, heart failure. Casein kinase-2 interacting protein-1 (CKIP-1) is an important regulator of pathological cardiac remodeling. However, the role of CKIP-1 in physiological cardiac hypertrophy is unknown. We subjected wild-type (WT) mice to a swimming exercise program for 21 days, which caused an increase in myocardial CKIP-1 protein and mRNA expression. We then subjected CKIP-1 knockout (KO) mice and myocardial-specific CKIP-1-overexpressing mice to the 21-day swimming exercise program. Histological and echocardiography analyses revealed that CKIP-1 KO mice underwent pathological cardiac remodeling after swimming, whereas the CKIP-1-overexpressing mice had a similar cardiac phenotype to the WT controls. Histone deacetylase 4 (HDAC4) is a key molecule in the signaling cascade associated with pathological hypertrophy; the phosphorylation levels of HDAC4 were markedly higher in CKIP-1 KO mouse hearts after the swimming exercise program. The phosphorylation levels of HDAC4 did not change after swimming in the hearts of CKIP-1-overexpressing or WT mice. Our results indicate that swimming, a mechanical stress that leads to physiological hypertrophy, triggers pathological cardiac remodeling in CKIP-1 KO mice. CKIP-1 is necessary for physiological cardiac hypertrophy *in vivo*, and for modulating the phosphorylation level of HDAC4 after physiological stress. Genetically engineering CKIP-1 expression affected heart health in response to exercise.

## Introduction

Pathological cardiac hypertrophy is characterized by depressed heart function, cardiac fibrosis, apoptosis, and ectopic expression of fetal cardiac genes such as atrial natriuretic peptide (ANP) and brain natriuretic peptide (BNP) (Bernardo et al., 2010; Maillet et al., 2013; Nakamura and Sadoshima, 2018). Physiological cardiac hypertrophy differs from pathological cardiac hypertrophy at the functional, histological, and molecular levels; it is characterized by enhanced or normal cardiac function and increased heart weight, with no cardiac fibrosis (Adams et al., 2008; Bernardo et al., 2018). Physiological cardiac growth occurs during postnatal heart development, pregnancy, and exercise training (Hill and Olson, 2008). Cardiac pumping capacity is the most important biological factor for cardiorespiratory fitness and is greatly affected by exercise. Exercise training confers important benefits to the heart and can improve cardiac function in heart failure patients (Marchionni et al., 2003; Platt et al., 2015). However, exercise is a highly complex stimulus, and the molecular mechanisms driving physiological hypertrophy are currently unclear. Therefore, it is important to investigate the molecular mechanisms underlying cardio-protective effects in response to endurance exercise.

Several signaling pathways related to heart disease are required for cardiac adaptation after exercise training (Maillet et al., 2013). Among these, Akt1 and phosphoinositide 3-kinase are activated in both physiological and pathological cardiac atrophy; these pathways are necessary for physiological remodeling and protect against pathological events (DeBosch et al., 2006; McMullen et al., 2007; Kemi et al., 2008). Cbp/p300-interacting transactivator with glutamate/aspartate-rich carboxy-terminal domain-4 (CITED4) and CCAAT/enhancer binding protein-β are critical for regulating the transcription of genes associated with exercise-induced cardiac hypertrophy (Boström et al., 2010; Bezzerides et al., 2016). On the other hand, histone deacetylases (HDACs) play important roles in pathological cardiac remodeling. Class I HDACs (HDACs 1–3) increase the expression of hypertrophic genes, whereas class IIa HDACs (HDACs 4 and 5) suppress hypertrophy (Weeks and Avkiran, 2015). In our previous study, we discovered that casein kinase-2 interacting protein-1 (CKIP-1) regulates the HDAC4/myocyte enhancer factor-2 pathway and inhibits pathological cardiac remodeling (Ling et al., 2012, 2018). However, the role of HDAC4 in modulating cardiac phenotypes following physiological stress is currently unclear.

CKIP-1 is a highly conserved protein serine/threonine kinase that interacts with specific CK2α subunits and functions as a master regulator of multiple cardiovascular diseases (Bosc et al., 2000; Fu and Zhang, 2019). CKIP-1 plays an important anti-atherosclerotic role by regulating foam cell formation and cholesterol metabolism (Fan et al., 2019). In a previous study, we found that pathological cardiac hypertrophy was exacerbated in cardiac-specific CKIP-1 knockout (KO) mutants subjected to cardiac pressure overload, and that overexpressing CKIP-1 protected against these effects (Ling et al., 2012). Furthermore, microgravity-induced cardiac atrophy was inhibited in mice overexpressing myocardial CKIP-1 (CKIP-1 TG mice) (Ling et al., 2018). Nevertheless, the regulatory mechanisms behind CKIP-1-mediated physiological cardiac hypertrophy are still unclear. In this study, we found that CKIP-1 is necessary for maintaining heart health in response to exercise. The CKIP-1 KO mice were unable to maintain normal or enhanced cardiac function following a 21-day swimming exercise program, and exhibited dysregulated cardiac homeostasis. Furthermore, the CKIP-1 KO mice underwent pathological cardiac remodeling and exhibited increased levels of HDAC4 phosphorylation following the swimming exercise program. The physiological cardiac hypertrophy was comparable between wild-type (WT) and cardiac-specific CKIP-1 TG mice after swimming.

## Materials and Methods

### Animals

The experimental procedures in mice and protocol complied with the National Institutes of Health Guidelines on the Use of Laboratory Animals and were approved by the Animal Care and Use Committee of China Astronaut Research and Training Center (ACC-IACUC-2020-002). Myocardial-specific CKIP-1 transgenic mice and CKIP-1-deficient mice have been obtained in previous reports (Ling et al., 2012). Male mice aged 8–10 weeks (weight 21–25 g) of WT, CKIP-1 transgenic (TG) mice, and CKIP-1 knockout (KO) mice were used in the study.

### Swimming Exercise Model

We followed the previously described 3-week swimming protocol to induce physiological cardiac hypertrophy (Wilkins et al., 2004; Taniike et al., 2008; Heineke et al., 2010). It began with 8 days of training time before the formal swimming exercise that was increased gradually 10 min every day until two 90-min phases were finished on the ninth day. Subsequently, mice went on swimming for as long as 12 additional days (21 days total). Of note, swimming training on the first day included two 10-min sessions separated by at least 4 h. Rest mice served as controls, which did not undergo any swimming training.

### Transthoracic Echocardiography

Mice were anesthetized with isoflurane (EZVET, No.04574). Two-dimensional guided M-mode echocardiography was performed using a high-resolution imaging system (Vevo 1100, Visual-Sonics Inc., Toronto, ON, Canada). Two-dimensional images are recorded in parasternal long- and short-axis projections with guided M-mode recordings at the midventricular level in both views. Left ventricular fractional shortening (FS) and ejection fraction (EF), and other parameters, including end-diastolic left ventricular posterior wall thickness (LVPWd), end-systolic left ventricular posterior wall thickness (LVPWs), end-diastolic left ventricular anterior wall thickness (LVAWd), end-systolic left ventricular anterior wall thickness (LVAWs), end-diastolic left ventricular internal diameters (LVIDd), end-systolic left ventricular internal diameters (LVIDs), and left ventricular mass were determined. Structure and function of left ventricular were measured as previously described (Ling et al., 2012).

### Histomorphological Examination

Myocardial tissues from the left ventricular of mice were harvested and fixed with 4% paraformaldehyde solution for 24 h. Then, tissues were paraffin embedded and sectioned serially according to routine procedures. The sections (4–5 μm) underwent hematoxylin-eosin (H&E) staining in preparation for histological staining and Masson’s trichrome staining to detect collagen deposition.

### Western Blot Analysis

Heart tissues were lysed in lysis buffer (50 mM Tris, pH7.5, 250 mM NaCl, 0.1% SDS, 2 mM dithiothreitol, 0.5% NP-40, 1 mM PMSF and protease inhibitor cocktail) on ice for 20 min. Equivalent protein lysates were used for 10% SDS-PAGE according to the molecular weight of target proteins, electro-transferred onto nitrocellulose membranes (Millipore, Billerica, MA, United States), and then blocked in 5% non-fat dry milk in TBST (10 mM Tris-Cl, 150 mM NaCl, 0.05% Tween-20, pH 7.5) for 2 h. Subsequently, the membrane was incubated overnight at 4°C with primary antibodies against HDAC4 (1:2,000, Cell Signaling Technology, United States, 5392S), p-HDAC4 (S632) (1:2,000, Cell Signaling Technology, United States, 3424S), p-HDAC4 (S246) (1:2,000, Cell Signaling Technology, United States, 3443S), CKIP-1 (1:2,000, Proteintech, United States, 24883-1), and GAPDH (1:5,000, Santa Cruz Biotechnology, United States, sc-25778). After incubating with the corresponding secondary antibodies conjugated to horseradish peroxidase (HRP), these were visualized using a chemiluminescence kit (Thermo Pierce, United States, No.32 109). The intensities of bands were analyzed with ImageJ software (NIH).

### Reverse-Transcription Polymerase Chain Reaction

Total mRNA was isolated from left ventricular tissues with TRIzol reagent (Takara, Japan) and reverse transcribed into cDNA with a Transcriptor First Strand cDNA Synthesis kit (Takara, Japan). The relative expression of specific genes was detected using quantitative real-time PCR with a SYBR Green PCR kit (Takara, Japan) in a Light Cycler (Eppendorf, Germany). The mRNA level of each gene was normalized to *Gapdh*, which served as an internal reference. The sequences of the primers are:

Mouse-ANP sense primer: 5′-TTCGGGGGTAGGAT TGACAG-3′,
Mouse-ANP anti-sense primer: 5′-CACACCACAAGGGC TTAGGA-3′,
Mouse-BNP sense primer: 5′-TGTTTCTGCTTTTCCTT TATCTG-3′,
Mouse-BNP anti-sense primer: 5′-TCTTTTTGGGTGTTC TTTTGTGA-3′,
Mouse-*Gapdh* sense primer: 5′-ACTCCACTCACGGC AAATTCA-3′,
Mouse- *Gapdh* anti-sense primer: 5′-GGCCTCACCCCAT TTGATG-3′,
Mouse-β-MHC sense primer: 5′-AGGCAAAGAAAGGC TCATCC-3′,
Mouse-β-MHC anti-sense primer: 5′-TGGAGCGCAAGTTT GTCATA-3′,
Mouse-CKIP-1 sense primer: 5′-CCGGATGGAAACCAT CAGTCT-3′,
Mouse-CKIP-1 anti-sense primer: 5′-TCAGCACCACATAG CGGTTT-3′.

### Statistical Analyses

Data are presented as mean ± SEM per experimental condition. Statistical differences between two groups were determined by the unpaired two-tailed Student’s *t*-test. Statistical differences among groups were analyzed by two-way analysis of variance (ANOVA) followed by the Bonferroni procedure. All the statistical tests are analyzed by Prism software (Graphpad prism for Windows, version 6.01) and SPSS (Version 14.0). Differences were considered statistically significant when *P* value was <0.05.

## Results

### Casein Kinase-2 Interacting Protein-1 Expression Changes in the Hearts of Mice Subjected to a 21-Day Swimming Exercise Program

**FIGURE 1:**
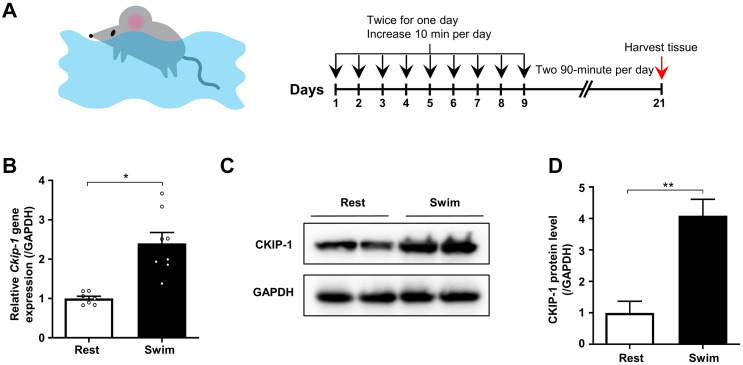
CKIP-1 expression changes in the hearts of mice after 21 days swimming exercise. **(A)** Schematic representation of swimming exercise induced physiological cardiac hypertrophy model in mice. **(B)** CKIP-1 mRNA levels assessed by quantitative polymerase chain reaction in mouse heart tissues from WT mice with 21 days swimming exercise, normalized to GAPDH. *n* = 7 replicates from rest mice and *n* = 8 replicates from swimming mice. **(C)** Western blot analysis of CKIP-1 expression in total protein extracts from rest and swimming exercise hearts with 21 days training (*n* = 3). **(D)** Quantification of CKIP-1 protein levels. Values are means ± SEM, ^∗^*P* < 0.05, ^∗∗^*P* < 0.01. Statistical differences between two groups were determined by the unpaired two-tailed Student’s t-test. CKIP-1, casein kinase-2 interacting protein-1; WT, wild type; GAPDH, glyceraldehyde 3-phosphate dehydrogenase.

To investigate whether CKIP-1 expression was altered during physiological cardiac hypertrophy, we subjected WT mice to a high-intensity swimming exercise regime (Figure 1A). Swimming exercise training remarkably promotes physiological cardiac growth. In comparison with the rest mice, trained mice had higher heart weight-to-body weight ratios (Supplementary Figure 1A) and cardiac contractile function (ejection fractions and fraction shortening; Supplementary Figures 1B,C). Furthermore, exercise training does not affect pathological hypertrophy marker genes atrial natriuretic peptide ANP, brain natriuretic peptide BNP, and β-MHC expression (Supplementary Figures 1D–F).

We measured the mRNA and protein levels of CKIP-1 in the hearts of mice after the swimming exercise via quantitative real-time polymerase chain reaction (qRT-PCR) and Western blot analyses, respectively. As shown in Figure 1B, *Ckip-1* mRNA levels were significantly higher in the hearts of WT mice after 21 days of swimming exercise compared with resting control mice. Similarly, CKIP-1 protein levels were significantly higher in the hearts of mice subjected to swimming exercise compared with resting mice (Figures 1C,D). These results suggest a potential role for CKIP-1 in the progression of swim-induced physiological cardiac hypertrophy.

### Physiological Stress Induced by Swimming Exercise Triggers Pathological Cardiac Remodeling in Casein Kinase-2 Interacting Protein-1 Knockout Mice

**FIGURE 2:**
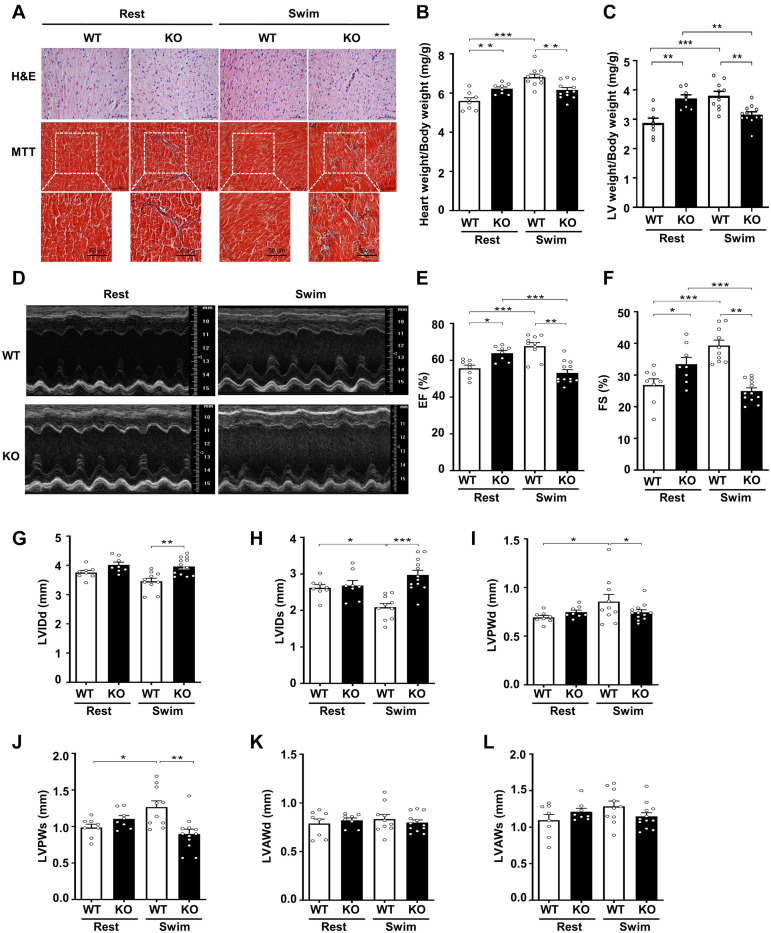
Physiological stress-swimming exercise causes pathological cardiac remodeling in CKIP-1-deficient mice. **(A)** Top, representative images of hematoxylin and eosin staining in hearts from WT and CKIP-1 KO aged (2-month-old) mice after rest or swimming exercise. Scale bars, 50 μm. Bottom, representative images of heart sections stained with Masson’s trichrome. Scale bars, 50 μm. **(B)** Heart weight (HW) to body weight (BW) ratios in WT and CKIP-1 KO mice with rest or swimming exercise. **(C)** Quantification of LV weight/body weight. **(D–F)** Cardiac function was monitored via echocardiography at 3 weeks after swimming exercise: **(D)** representative M-mode echocardiographic images of each study group at 3 weeks; **(E)** ejection fraction (EF) in WT-Rest, KO-Rest, WT-Swimming, and KO-Swimming mice; **(F)** fractional shortening (FS) in WT and CKIP-1 KO mice after rest or swimming exercise. **(G–L)** Quantitative analysis of the diastolic and systolic left ventricular internal diameter (LVIDd and LVIDs), LV posterior wall thickness (LVPWd and LVPWs), and LV anterior wall thickness (LVAWd and LVAWs) from WT and KO mice by echocardiography after swimming exercise. Values are means ± SEM, *n* = 8∼12, ^∗^*P* < 0.05, ^∗∗^*P* < 0.01, ^∗∗∗^*P* < 0.001. Statistical differences among groups were analyzed by two-way analysis of variance (ANOVA) followed by the Bonferroni procedure. CKIP-1, casein kinase-2 interacting protein-1; WT, wild type; KO, knockout; LV, left ventricular.

Next, we investigated the role of CKIP-1 in the regulation of heart growth after swimming exercise. Eight-week-old CKIP-1 KO mice and littermate controls were subjected to a swimming protocol to induce physiological cardiac hypertrophy. CKIP-1 KO mice exhibited cardiac fibrosis prior to exercise, which was significantly exacerbated after the 21-day swimming exercise program, as revealed by Masson trichrome staining of cardiac tissues and quantification of interstitial fibrosis (Figure 2A and Supplementary Figure 2A). The WT mice exhibited a remarkable hypertrophic response to the exercise stimuli; however, the heart weight to body weight ratio and left ventricular (LV) weight to body weight ratio were significantly lower in CKIP-1 KO mice after the swimming exercise (Figures 2B,C).

To further evaluate the effects of CKIP-1 ablation on cardiac function and structure following swimming exercise, we performed echocardiography on CKIP-1-TG and WT mice. Notably, CKIP-1 KO mice exhibited signs of cardiac dysfunction after swimming, such as decreased ejection fractions and fraction shortening, while WT mice displayed significant increases in cardiac function (Figures 2D–F). Swim-trained WT mice exhibited attenuated LV end-systolic internal diameters (LVIDs). Furthermore, the diastolic and systolic LV internal diameters (LVIDd and LVIDs, respectively) were significantly larger in the hearts of CKIP-1 KO mice after swimming compared with WT mice (Figures 2G,H). The diastolic and systolic left ventricular posterior walls (LVPWd and LVPWs, respectively) were significantly thicker in the hearts of WT mice after the swimming exercise compared with before; however, this hypertrophy phenotype was not present in CKIP-1 KO mice, which exhibited no significant difference in LVPWd or LVPWs thickness after the swimming exercise (Figures 2I,J). No significant difference in the LV end-diastolic anterior wall thickness (LVAWd) (Figure 2K) or LV end-systolic anterior wall thickness (LVAWs) (Figure 2L) was observed between the WT and CKIP-1 KO mice after swimming, whereas cardiac stroke volume (SV) was drastically reduced in CKIP-1 KO mice after swimming (Supplementary Figure 2B). Meanwhile, heart rate (HR) was also depressed in the hearts of CKIP-1 KO mice with swimming exercise (Supplementary Figure 2C). Our findings suggest that CKIP-1 KO mice underwent maladaptive cardiac remodeling in response to swimming exercise, leading to attenuated cardiac function and fibrosis.

### Myocardial Casein Kinase-2 Interacting Protein-1 Overexpression Does Not Affect Physiological Cardiac Remodeling in Response to Swimming Exercise

**FIGURE 3:**
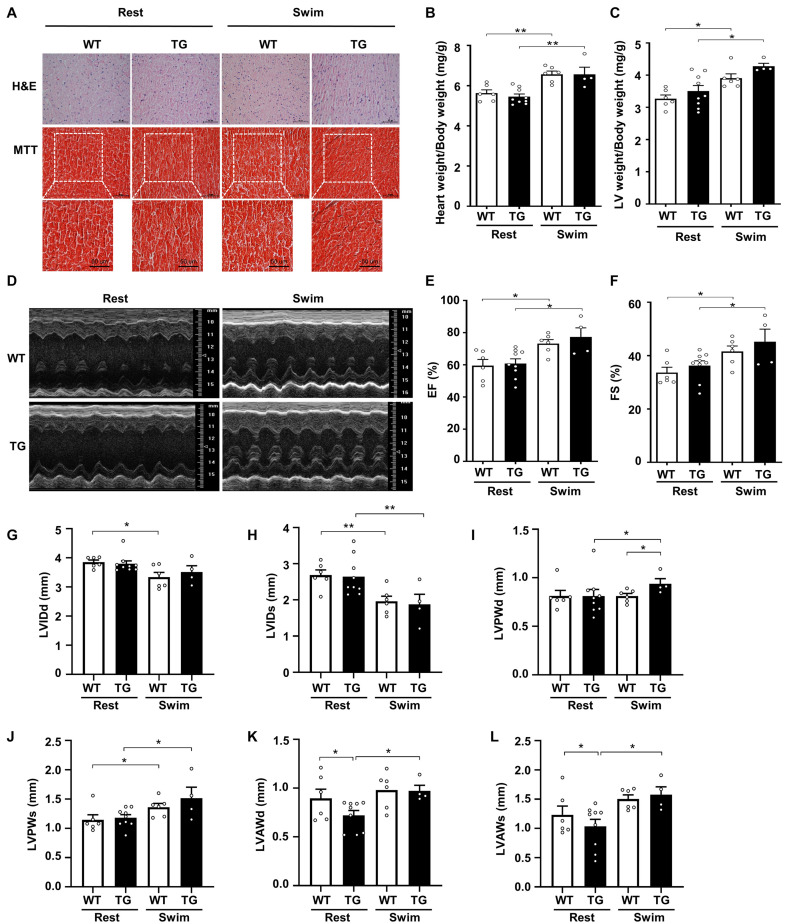
Myocardial CKIP-1 overexpression does not affect physiological cardiac remodeling in response to swimming exercise. **(A)** CKIP-1 TG mice and WT littermates at 2 months of age were subjected to rest or swimming exercise. Histological sections from hearts were stained with H&E (scale bars, 50 μm) and MTT to detect fibrosis (scale bars, 50 μm). The ratios of heart weight to body weight **(B)** and LV mass to body weight **(C)** in WT and CKIP-1 TG mice after swimming exercise. Representative echocardiographic M-mode images **(D)**, ejection fraction **(E)**, and fractional shortening **(F)** evaluated by echocardiography in anesthetized WT and CKIP-1-TG mice with rest or swimming exercise. **(G–L)** Transthoracic echocardiography evaluating the left ventricular structure of WT and TG mice following swimming. Values are means ± SEM, *n* = 4∼9, ^∗^*P* < 0.05, ^∗∗^*P* < 0.01. Statistical differences among groups were analyzed by two-way analysis of variance (ANOVA) followed by the Bonferroni procedure. CKIP-1, casein kinase-2 interacting protein-1; WT, wild type; TG, transgenic; H&E, hematoxylin and eosin; MTT, Masson’s trichrome.

To further explore the physiological function of cardiac CKIP-1 *in vivo* after swimming, we subjected cardiac-specific CKIP-1-TG transgenic mice and littermate controls to a swimming exercise program. We evaluated baseline cardiac morphology and hypertrophy prior to exercise. CKIP-1 TG mice showed no differences in individual cardiomyocyte size, cardiac fibrosis (Supplementary Figure 3A), and heart weight-to-body weight ratio or LV weight/body weight compared with the controls (Figures 3A–C). Moreover, the left ventricular ejection fractions and fraction shortening were not significantly different between CKIP-1 TG mice and controls. These results indicate a lack of adverse cardiac remodeling at baseline in CKIP-1 TG mice (Figures 3D–F). After the swimming program, an increase in hypertrophic growth and cardiac function was observed in CKIP-1 TG mice, similar to the WT controls.

Echocardiographic measurements revealed a reduction in the LVIDd and LVIDs after swimming in both CKIP-1 TG and WT mice (Figures 3G,H). The LVPWd was thicker in the hearts of swim-trained CKIP-1 TG mice compared with swim-trained WT hearts and CKIP-1 TG resting controls (Figure 3I). Compared with the resting controls, the LVPWs was significantly thicker in the hearts of CKIP-1 TG and WT mice following swimming exercise (Figure 3J). The diastolic and systolic LV anterior wall thickness (LVAWd and LVAWs) increased after the swimming program in the hearts of CKIP-1 TG mice, but were lower in CKIP-1 TG mice compared with the controls (Figures 3K,L). Stroke volume (SV) and heart rate (HR) were unchanged in CKIP-1 TG compared with WT groups after swimming (Supplementary Figures 3B,C). Taken together, these findings indicated that cardiac-specific overexpression of CKIP-1 in adult mice induced physiological cardiac hypertrophy, with increased heart weight and cardiac function without interstitial fibrosis after swimming.

### Casein Kinase-2 Interacting Protein-1 Regulates the Phosphorylation of Histone Deacetylase 4 During Swimming Exercise-Induced Physiological Cardiac Hypertrophy

**FIGURE 4:**
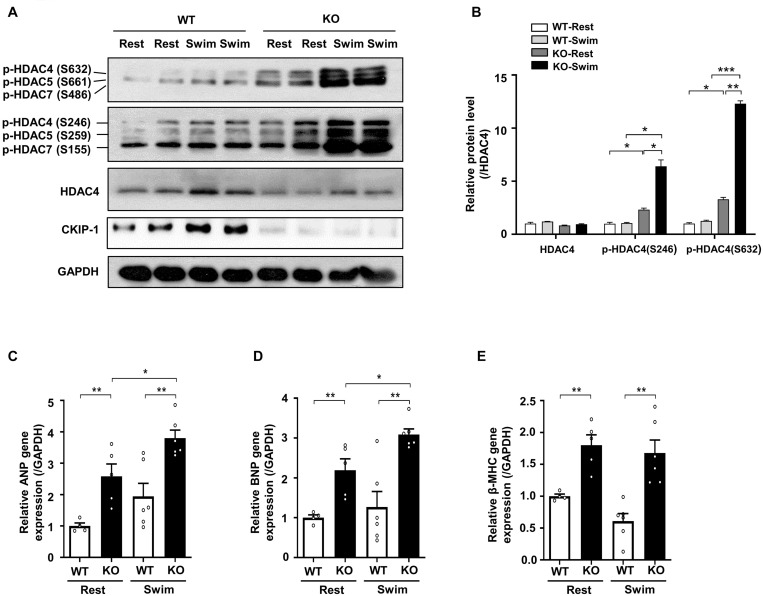
CKIP-1 deficiency exacerbates HDAC4 phosphorylation during exercise induced physiological cardiac hypertrophy. CKIP-1, phosphorylated-HDAC4 (S632), phosphorylated-HDAC4 (S246), and HDAC4 protein expression were measured by Western blot **(A)** and quantification of p-HDAC4 (S632), p-HDAC4 (S246), and HDAC4 levels in WT and CKIP-1 KO mice in response to swimming exercise **(B)**. **(C–E)** Quantitative polymerase chain reaction analyses of the mRNA levels of hypertrophic marker genes (ANP, BNP, and β-MHC) in the indicated groups. Values are means ± SEM, *n* = 4∼6, ^∗^*P* < 0.05, ^∗∗^*P* < 0.01, ^∗∗∗^*P* < 0.001. Statistical differences among groups were analyzed by two-way analysis of variance (ANOVA) followed by the Bonferroni procedure. CKIP-1, casein kinase-2 interacting protein-1; WT, wild type; KO, knockout; HDAC4, histone deacetylase 4; ANP, atrial natriuretic peptide; BNP, brain natriuretic peptide; β-MHC, myosin heavy chain β.

**FIGURE 5:**
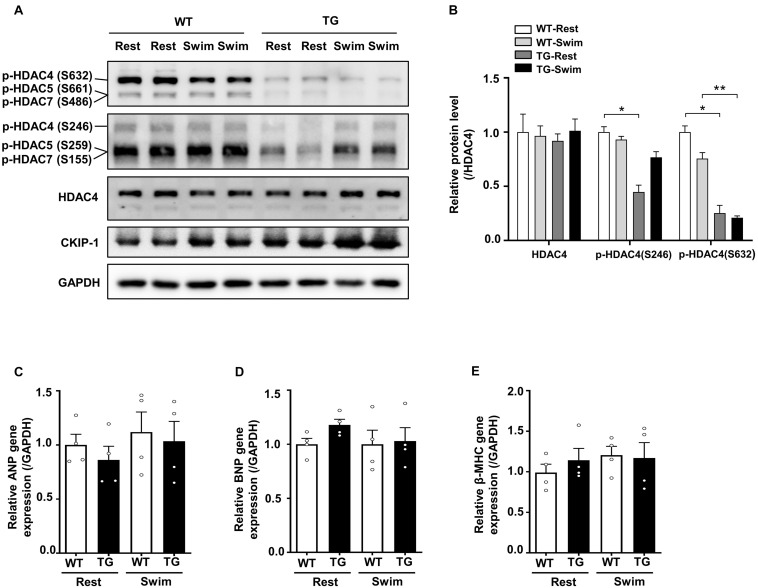
Overexpression of CKIP-1 inhibits the phosphorylation of HDAC4. **(A)** Western blotting showing the expression levels of CKIP-1, phosphorylated HDAC4 (S632), phosphorylated HDAC4 (S246), and the total protein expression levels of HDAC4. **(B)** Quantification of p-HDAC4 (S632), p-HDAC4 (S246), and HDAC4 levels. **(C–E)** Transcript levels of hypertrophic marker genes (ANP, BNP, and β-MHC) in the hearts of WT and CKIP-1 TG mice after swimming exercise. Values are means ± SEM, *n* = 4∼6, ^∗^*P* < 0.05, ^∗∗^*P* < 0.01. Statistical differences among groups were analyzed by two-way analysis of variance (ANOVA) followed by the Bonferroni procedure. CKIP-1, casein kinase-2 interacting protein-1; WT, wild type; TG, transgenic; HDAC4, histone deacetylase 4; ANP, atrial natriuretic peptide; BNP, brain natriuretic peptide; β-MHC, myosin heavy chain β.

**FIGURE 6:**
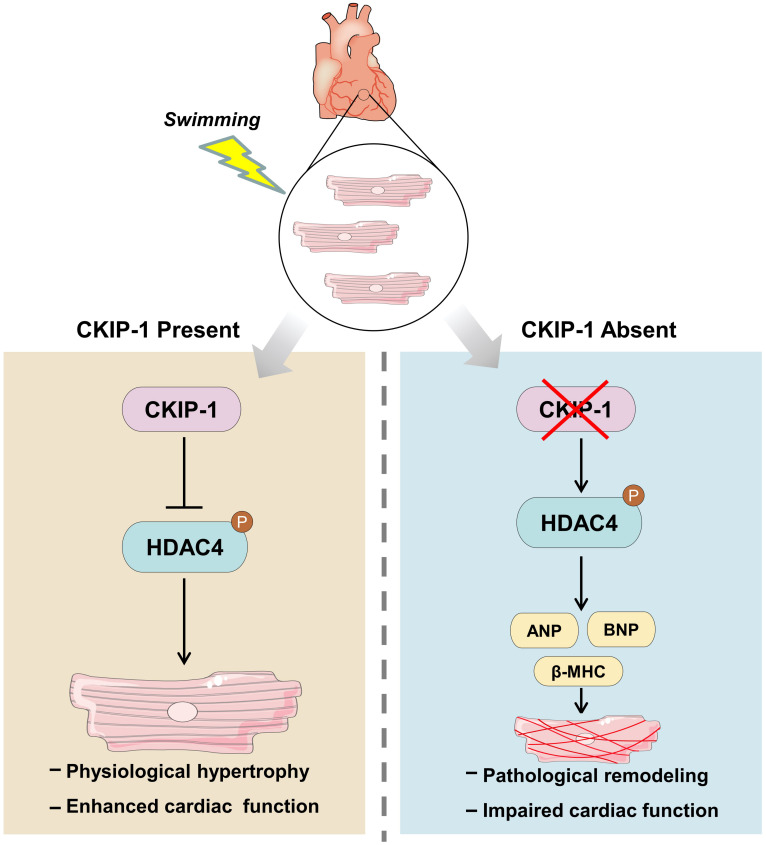
Model for the mechanism of CKIP-1 regulation of cardiac remodeling by inhibition of HDAC4 phosphorylation during swimming.

Our previous studies demonstrated that CKIP-1 plays a key role in the suppression of cardiac hypertrophy by promoting HDAC4 dephosphorylation (Ling et al., 2012, 2018). CKIP-1 deficiency triggers translocation of HDAC4 from the nucleus into the cytoplasm in cardiomyocytes (Supplementary Figure 4). To better understand the cellular signaling pathways contributing to the CKIP-1 KO and CKIP-1 TG phenotypes after the swimming exercise, we examined the phosphorylation levels of HDAC4 in heart tissues. HDAC4 phosphorylation at Ser246 and Ser632 was significantly higher in CKIP-1 KO mice compared with WT mice, both at rest and during swimming exercise. HDAC4 activation was further increased in CKIP-1 KO mice after the 21-day swimming exercise (Figures 4A,B); HDAC5 phosphorylation levels were also higher after exercise (Supplementary Figure 5). Canonical heart failure-associated marker genes, including ANP, BNP, and myosin heavy chain β (β-MHC), were upregulated in the hearts of CKIP-1 KO mice compared with controls under both baseline and swim-stimulated conditions (Figures 4C–E). In contrast to the CKIP-1 KO mice, the Ser246 and Ser632 HDAC4 phosphorylation levels in the hearts of CKIP-1 TG mice were similar before and after swim training, and lower than in the WT controls (Figures 5A,B). qRT-PCR analysis revealed that the pathological hypertrophic markers (ANP, BNP, β-MHC) were not significantly different (Figures 5C–E). Together, these results indicated that CKIP-1 KO mice promoted HDAC4 activation and reactivation of the fetal gene program in adult hearts in response to swimming exercise, thereby stimulating pathological cardiac remodeling. On the other hand, myocardial-specific CKIP-1 overexpression did not affect HDAC4 phosphorylation levels following swimming exercise. Indeed, cardiac HDAC4 phosphorylation levels were lower in CKIP-1 TG mice compared with WT controls. No significant differences in the expression of pathological hypertrophic marker genes were observed after swimming exercise in CKIP-1 TG mice (Figure 6).

## Discussion

In this study, we found that CKIP-1 expression was upregulated in response to exercise stimuli, and was required for adaptive cardiomyocyte growth following swimming exercise. Exercise causes electrical, structural, and functional changes in the heart, which result in an increase in blood ejection force (Naud et al., 2010; D’Andrea et al., 2015). The benefits of exercise on cardiac health include reduced risk of cardiovascular disease risk and greater longevity (Sharma et al., 2015; Moreira et al., 2020). Notably, we found that CKIP-1 KO mice underwent pathological cardiac remodeling after 21 days of swimming exercise, characterized by increased ventricular fibrosis and cardiac dysfunction. By contrast, cardiac-specific CKIP-1-TG mice exhibited normal physiological cardiac hypertrophy after swimming exercise. We then investigated the molecular mechanisms mediating the cardiac phenotypes of CKIP-1 KO and TG mice after swimming exercise, and found that CKIP-1 was involved in the regulation of HDAC4 phosphorylation at Ser246 and Ser632. Our results suggest that CKIP-1 KO activated HDAC4 phosphorylation and fetal gene expression, which led to maladaptive cardiac remodeling. Moreover, exercise did not trigger cardiac improvement in CKIP-1 KO mice, which instead exhibited a pathological response to exercise stimuli. Taken together, our data suggests that CKIP-1 is essential for healthy adaptive cardiac growth in response to swimming exercise.

Exercise leads to physiological cardiac hypertrophy and protects the heart from pathological growth (Sharma and Pelliccia, 2020). In response to exercise, a number of physiological changes take place that enable the heart to pump more blood per minute, thus, greatly increasing cardiac output (Heiss et al., 1976; Heinonen et al., 2015). In response to cardiac pressure overload, the myocardial wall thickness increases, without any effect on chamber diameter, to increase blood flow (Pelliccia et al., 1990). Cardiac morphology and structure change profoundly after exercise; the hearts of elite athletes that participate in regular endurance training are markedly larger than those of the general population, but this is not associated with fibrosis (Lovic et al., 2017). Our finding that cardiac mass was augmented without fibrosis in CKIP-1 TG mice after swimming exercise is consistent with previous studies. Echocardiography analyses of cardiac structure and function indicated a significant increase in LV wall thickness and cardiac function after swimming in mouse hearts overexpressing CKIP-1. CKIP-1 KO mice displayed pathological cardiac phenotypes following swimming exercise. In summary, we demonstrated that CKIP-1 is a key regulator of exercise-induced physiological cardiac growth.

Cardiac hypertrophy can be pathological or physiological. Pathological hypertrophy is associated with altered cardiac gene expression, fibrosis, cardiac dysfunction, and increased morbidity and mortality, whereas physiological hypertrophy is characterized by a normally organized cardiac structure and normal or enhanced cardiac function (Wilkins et al., 2004; Kong et al., 2005). The mechanisms by which divergent signaling mechanisms can lead to distinct patterns of pathological and physiological cardiac hypertrophy are still unknown to cardiac biologists (Wilkins et al., 2004; Heineke and Molkentin, 2006; Liu et al., 2009). Cardiomyocyte-specific deletion of CITED4 in mice causes maladaptive remodeling and functional deficit in response to endurance exercise (Lerchenmüller et al., 2020). Notably, this phenotype differs from typical physiological hypertrophy. Similarly, CKIP-1 KO mice also demonstrated a pathological phenotype after exercise stimuli, characterized by interstitial fibrosis accumulation, increased expression of pathological-related genes, and reduced systolic function.

The mammalian heart undergoes significant remodeling after exercise training (Lim et al., 2012). Investigating ways to exploit the beneficial effects of exercise is an exciting field of cardiac study. However, the mechanisms underlying exercise-induced cardiac protection are not fully understood. HDACs are divided into four major classes: class I–III sirtuins (SIRT1, SIRT2, and SIRT3) and class IV HDACs (Menzies et al., 2016; Li et al., 2020). Given that HDACs are involved in many cellular signaling pathways and diseases, compounds that can inhibit the activity of these enzymes possess therapeutic potential. Multiple HDAC inhibitors have been clinically tested as therapies for nervous system disorders and immunological diseases (Falkenberg and Johnstone, 2014). However, studies exploring the potential of HDAC inhibitors as heart disease treatments are still in their preliminary stages (Bush and McKinsey, 2010). Among the HDACs, classes II and III HDACs appear to have a protective role against cardiovascular disease (Kook et al., 2003; Kee et al., 2013), whereas class I HDACs protect against vessel injury but have detrimental effects on the heart (Kwon et al., 2016; Li et al., 2017). HDACs are therefore attractive targets for heart disease treatments. Moreover, CKIP-1 inhibits pathological cardiac remodeling by increasing HDAC4 dephosphorylation in transverse aortic constriction-induced and microgravity-induced cardiac atrophy models (Ling et al., 2012, 2018). In the present study, the CKIP-1–HDAC4 axis was found to be a crucial link between physiological and pathological growth in hearts. CKIP-1 KO mice activated HDAC4 phosphorylation after swimming exercise, leading to pathological cardiac remodeling. CKIP-1 TG mice displayed similar physiological cardiac hypertrophy to WT controls, and the HDAC4 phosphorylation levels in the hearts of CKIP-1 TG mice did not change under physiological stress. CKIP-1 TG reduced phosphorylation of HDACs, and then the HDACs translocated in the nucleus to inhibit MEF2 transcriptional activity. The inhibition of MEF2 transcriptional activity could suppress pathological cardiac remodeling, but had no effect on physiological cardiac hypertrophy.

This study revealed the critical role of CKIP-1 in the regulation of physiological cardiac growth. Our findings highlight the importance of HDAC4 signaling governed by CKIP-1 in mouse hearts during swimming exercise, providing insight into potential strategies for maintaining heart health.

## Data Availability Statement

The original contributions presented in the study are included in the article/Supplementary Material, further inquiries can be directed to the corresponding author/s.

## Ethics Statement

The animal study was reviewed and approved by the experimental procedures in mice and protocol complied with the National Institutes of Health Guidelines on the Use of Laboratory Animals and were approved by the Animal Care and Use Committee of China Astronaut Research and Training Center (ACC-IACUC-2020-002).

## Author Contributions

YZ and SL performed the majority of the experiments, analyzed data, and prepared the manuscript. GK provided us with technical support. GZ, YuL, and JL helped with the transthoracic echocardiography experiments. RD, XJ, DZ, and ZL provided suggestions for the project and critically reviewed the manuscript. SL, Y-ZC, and YiL supervised the project and the mauscript. All authors have read and agreed to the published version of the manuscript.

## Conflict of Interest

The authors declare that the research was conducted in the absence of any commercial or financial relationships that could be construed as a potential conflict of interest.
