# Supplementary material for: Casein Kinase-2 Interacting Protein-1 Regulates Physiological Cardiac Hypertrophy via Inhibition of Histone Deacetylase 4 Phosphorylation
Source: Front Physiol. 2021 Jun 15;12:678863. doi: 10.3389/fphys.2021.678863 (PMC8239235; doi:10.3389/fphys.2021.678863)

SUPPLEMENTAL MATERIAL

CKIP-1 regulates physiological cardiac hypertrophy through inhibition of HDAC4 phosphorylation

Yinlong Zhao[1, 2], Shukuan Ling[2*], Guohui Zhong[2, 3], Yuheng Li[2], Jianwei Li[2], Ruikai Du[2], Xiaoyan Jin[2], Dingsheng Zhao[2], Zizhong Liu[2], Guanghan Kan[2], Yan-Zhong Chang[1*], Yingxian Li[2*]

1. Key Laboratory of Molecular and Cellular Biology of Ministry of Education, College of Life Science, Hebei Normal University, Shijiazhuang, China.
2. State Key Laboratory of Space Medicine Fundamentals and Application, China Astronaut Research and Training Center, Beijing, China.
3. School of Aerospace Medicine, Fourth Military Medical University, Xi'an, China.

*** Correspondence**

Yingxian Li, PhD, State Key Laboratory of Space Medicine Fundamentals and Application, China Astronaut Research and Training Center, Haidian District, Beiqing Road, Beijing, China. E-mail: yingxianli@aliyun.com

Yan-Zhong Chang, Key Laboratory of Molecular and Cellular Biology of Ministry of Education, College of Life Science, Hebei Normal University, Yuhua District, 20, Nanerhuan Eastern Road, Shijiazhuang, China. E-mail: chang7676@163.com

Shukuan Ling, PhD, State Key Laboratory of Space Medicine Fundamentals and Application, China Astronaut Research and Training Center, Haidian District, Beiqing Road, Beijing, China. E-mail: sh2ling@126.com

Supplementary Figure 1: Swimming exercise training promotes physiological cardiac growth. **(A)** Heart weight (HW) to body weight (BW) ratios in rest and swim mice. **(B and C)** Echocardiographic assessment of ejection factor (EF) (B) and fractional shortening (FS) (C). **(D-F)** Relative mRNA expression of hypertrophic marker genes (ANP, BNP and β-MHC) in the hearts of WT mice after swimming exercise. Values are means ± SEM, n=7~8, ***P*<0.01, ****P*<0.001. Statistical differences between two groups were determined by the unpaired 2-tailed Student's t-test.

Supplementary Figure 2: Effect of swimming training on the exercise-induced cardiac growth program in CKIP-1 knockout mice. **(A)** Quantification of interstitial fibrosis in WT and CKIP-1 KO mice after swimming exercise. **(B and C)** Stroke volume (SV) and heart rate (HR) as measured by echocardiography in the mice. Values are means ± SEM, n=8~12, **P*<0.05, ****P*<0.001. Statistical differences among groups were analyzed by two-way analysis of variance (ANOVA) followed by the Bonferroni procedure.

Supplementary Figure 3: Effect of swimming training on the exercise-induced cardiac growth program in CKIP-1 transgenic mice. **(A)** Quantification of interstitial fibrosis in WT and CKIP-1 transgenic mice after swimming exercise. **(B and C)** Stroke volume (SV) and heart rate (HR) as measured by echocardiography in the mice. Values are means ± SEM, n=4~9. Statistical differences among groups were analyzed by two-way analysis of variance (ANOVA) followed by the Bonferroni procedure.

Supplementary Figure 4: CKIP-1 regulates HDAC4 translocation from the nucleus to the cytoplasm in CKIP-1 KO mice heart. Immunohistochemical staining with HDAC4 in CKIP-1 KO mice and WT littermates mice hearts. Scale bars: 10 μm.

Supplementary Figure 5: Expression of phospho-HDAC4/5/9 (Ser 246/259/220) are increased in CKIP-1 KO mice subjected to swimming exercise. Immunoblots showing the expression pattern of phospho-HDAC4/5/9 and HDAC5 in CKIP-1 KO mice and WT littermates mice cardiac tissues.

Supplemental Figure 1

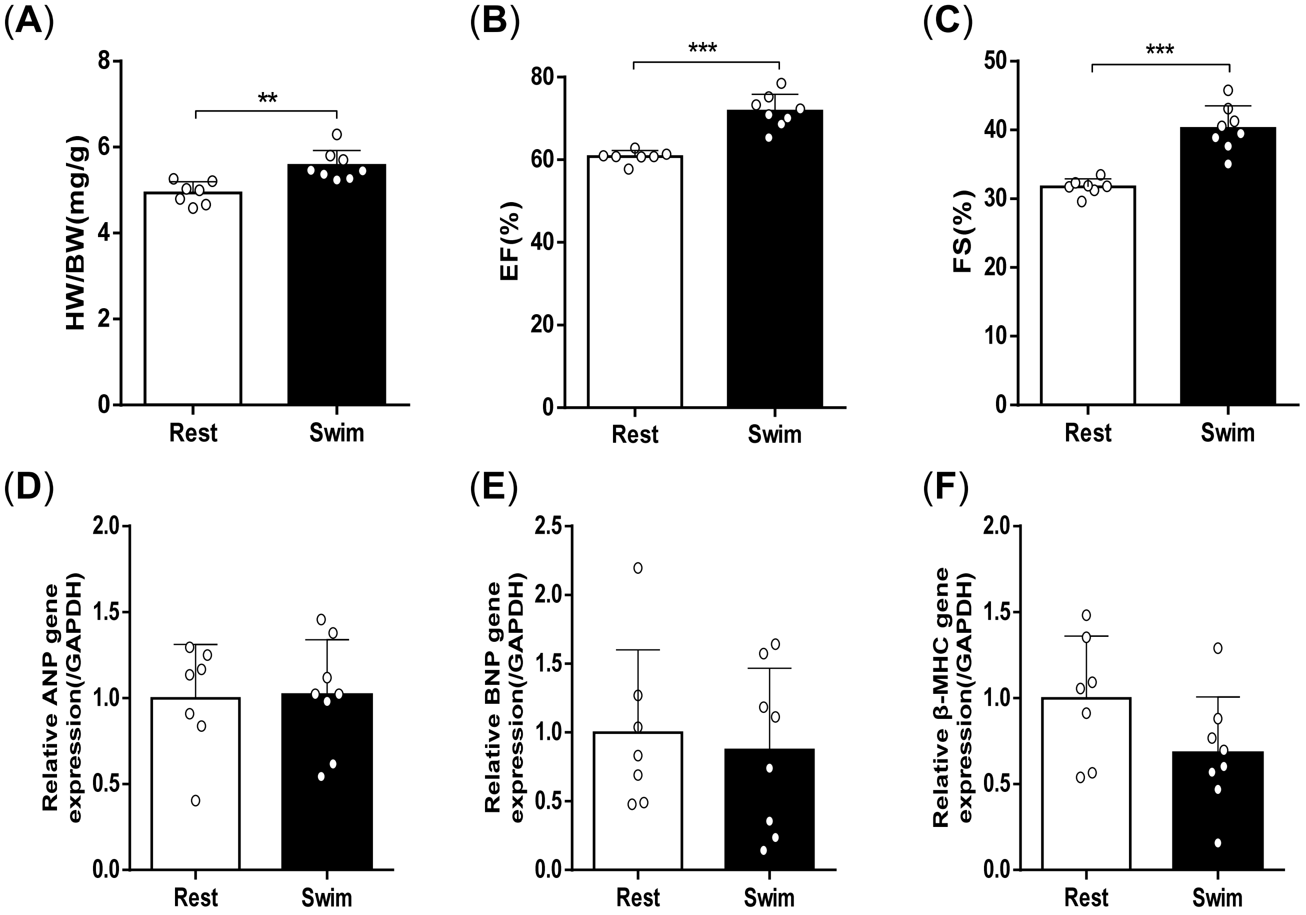

Supplemental Figure 2

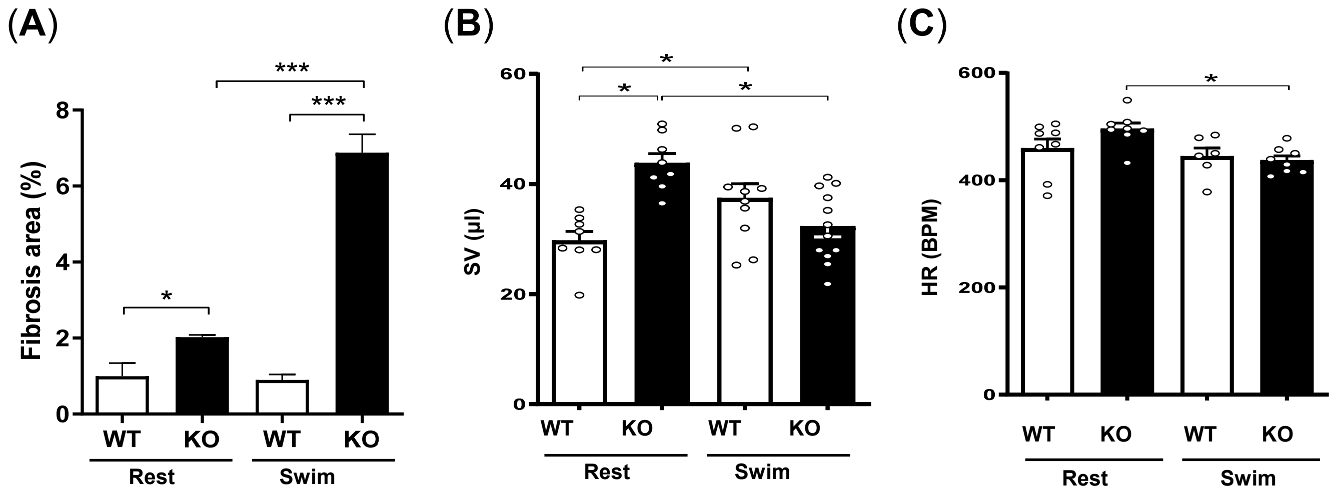

Supplemental Figure 3

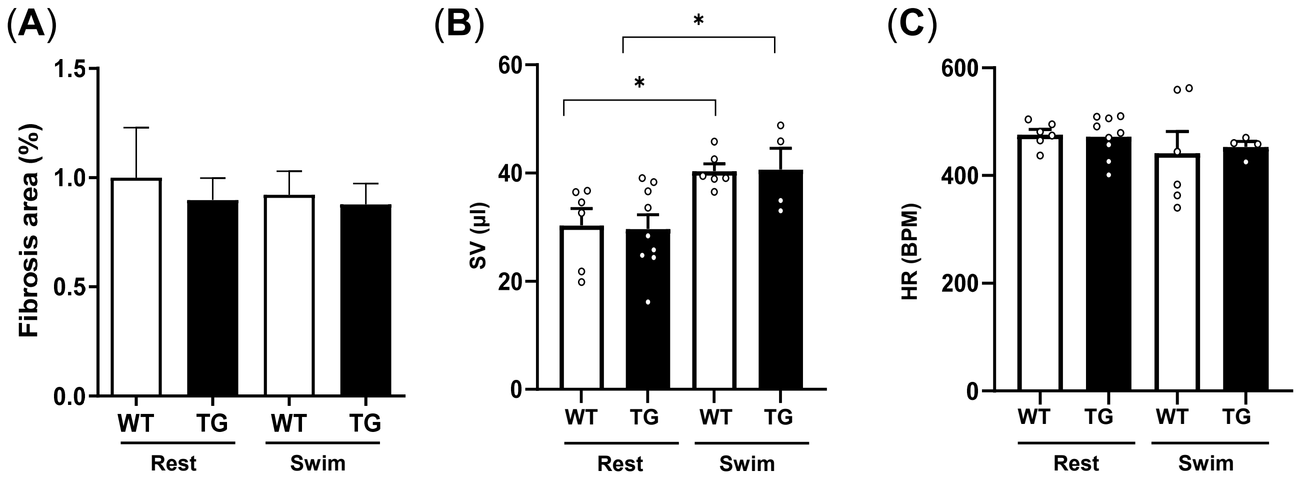

Supplemental Figure 4

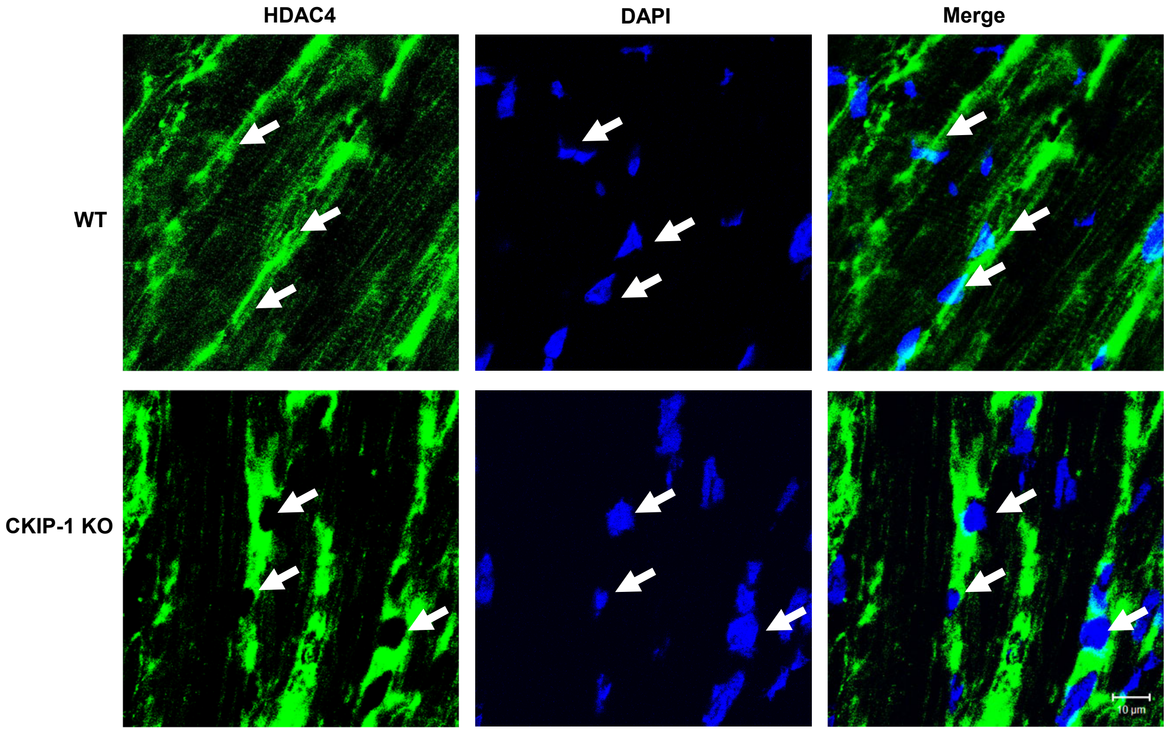

Supplemental Figure 5

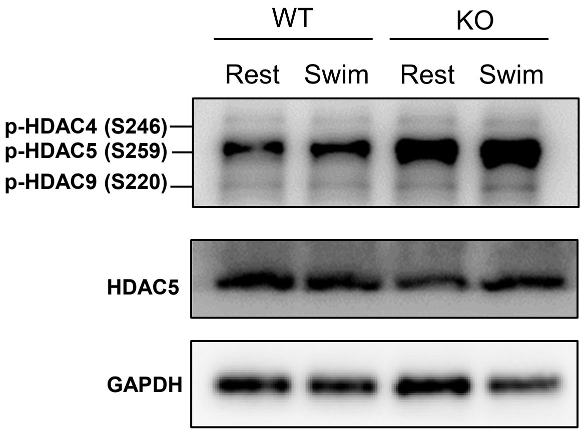

Supplement: Supplementary file 1 [file Data_Sheet_1.PDF]
